# Impairment of Kidney Function in Patients with Chronic Coronary Syndromes

**DOI:** 10.3390/jcm14186607

**Published:** 2025-09-19

**Authors:** Katarzyna Charkiewicz-Szeremeta, Emilia Sawicka-Śmiarowska, Danuta Czarnecka, Marlena Dubatówka, Zbigniew Gąsior, Tomasz Hryszko, Piotr Jankowski, Małgorzata Knapp, Dariusz A. Kosior, Aldona Kubica, Klaudia Mickiewicz, Andrzej Pająk, Marek Rajzer, Marek Styczkiewicz, Renata Wolfshaut-Wolak, Karol A. Kamiński

**Affiliations:** 1 Department of Population Medicine and Lifestyle Diseases Prevention, Medical University of Bialystok, 15-269 Bialystok, Poland; katarzyna.charkiewicz006@gmail.com (K.C.-S.);; 2 Department of Cardiology and Internal Medicine, Center of Postgraduate Medical Education, Grochowski Hospital, 04-073 Warszawa, Poland; 3 Department of Cardiology and Internal Diseases, Medical University of Bialystok, 15-276 Bialystok, Poland; 4 1st Department of Cardiology, Interventional Electrocardiology and Hypertension, Institute of Cardiology, Jagiellonian University Medical College, 30-688 Kraków, Poland; 5 Department of Cardiology, Medical University of Silesia, 40-635 Katowice, Poland; 6 2nd Department of Nephrology, Hypertension, and Internal Medicine with Dialysis Unit, Medical University of Bialystok, 15-276 Bialystok, Poland; 7 Department of Internal Medicine and Geriatric Cardiology, Center of Postgraduate Medical Education, 00-416 Warszawa, Poland; 8 Department of Epidemiology and Health Promotion, School of Public Health, Center of Postgraduate Medical Education, 01-826 Warszawa, Poland; 9 Mossakowski Medical Research Institute, Polish Academy of Science, 02-106 Warszawa, Poland; 10 Department of Sports Medicine, Center of Postgraduate Medical Education, 01-813 Warszawa, Poland; 11 Department of Cardiac Rehabilitation and Health Promotion, Collegium Medicum, Nicolaus Copernicus University, 85-067 Bydgoszcz, Poland; 12 Department of Epidemiology and Population Studies, Jagiellonian University Medical College, 31-066 Kraków, Poland; 13 Department of Cardiology, Jan Paweł II Hospital, 22-400 Zamość, Poland; 14 Institute of Nursing and Midwifery, Jagiellonian University Medical College, 31-501 Kraków, Poland

**Keywords:** chronic coronary syndrome, urine albumin/creatin ratio, eGFR, KDIGO classification

## Abstract

**Background**: Kidney function is critical for cardiovascular health, and its appropriate assessment entails proper determination of prognosis in patients with chronic coronary syndromes (CCSs). However, assessment of the urinary spot albumin to creatinine ratio (uACR) is often overlooked, whereas it is crucial for determination of chronic kidney disease (CKD). This study assesses the prevalence of impaired kidney function in patients with CCS based on their eGFR and albuminuria. **Methods and results**: This study comprised a total of 1957 patients from seven regions in Poland, aged ≤ 80 years, who, 6–18 months earlier, were hospitalized for acute coronary syndrome or elective myocardial revascularization. Complete uACR and eGFR data were obtained from 1152 patients (median age was 67 years, and 71.23% of participants were male). The finding of albuminuria reclassified the CKD in 17% (200) patients, suggesting that a patient’s risk cannot be ascertained only based on their eGFR result. CKD reclassification by albuminuria was observed in older (*p* < 0.001) patients with higher BPs (*p* = 0.008), BPd (*p* = 0.038), HR (*p* < 0.001), fasting glucose (*p* < 0.001), and HbA1c (*p* < 0.001) and decreased HDL concentration (*p* = 0.001); hence, this is the population where uACR assessment is particularly valuable. **Conclusions**: In a notable percentage of patients with CCS, their kidney function classification is changed based on their albuminuria. Therefore, it is important to include albuminuria in the routine assessment of patients with cardiovascular disease.

## 1. Introduction

One of the leading causes of death worldwide and the first cardiovascular disease (CVD) is coronary artery disease (CAD), sometimes identified as “chronic coronary syndromes” (CCSs) to describe the whole spectrum of patients more accurately [1].

Coronary artery disease (CAD) and chronic kidney disease (CKD) are frequently associated. In approximately 20% of patients with a history of CAD, CKD is present [2]. These patients experience symptoms such as typical chest pain and pressure behind the breastbone and may also experience exertional dyspnea, nausea and epigastric pain, palpitations, and general weakness. Symptoms typical of chronic coronary syndromes may be masked, and, in patients with kidney disease, heart disease often manifests atypical symptoms, such as unusual shortness of breath, fatigue, or gastrointestinal symptoms, rather than the characteristic chest pain.

Therefore, it would be worth looking at this particular group of patients from a nephrologic perspective, and, since individuals with CKD are frequently excluded from research, data on patients with both CCS and CKD are difficult to analyze, especially with regard to interventions that may improve their prognosis [3].

Most reports indicate that CKD increases the risk of all-cause and CVD mortality; therefore, it is essential to analyze the prevalence of CKD both in the general population as well as in high risk individuals, such as people with CCS [4].

According to the European Society of Cardiology’s (ESC) 2021 guidelines on CVD prevention in clinical practice, accurate patient classification based on CVD risk, which affects preventive interventions, requires the evaluation of the estimated GFR (eGFR) and urine albumin to creatinine ratio (uACR). The presence of an increased albuminuria uACR ≥ 30 mg/g or an eGFR < 60 mL/min/1.73 m^2^ are the key diagnostic criteria for CKD in clinical practice [5]. Six eGFR and three uACR categories are used to stage chronic kidney disease [5]. Higher categories are related to a higher risk of unfavorable outcomes, such as fatal and non-fatal cardiovascular disease events. One of the main takeaways from the 2012 KDIGO guideline is that individuals with normal eGFR can still be diagnosed with CKD if their albuminuria is increased. A low eGFR is not a prerequisite for CKD diagnosis anymore. According to previous 2021 guidelines, CKD was considered a cardiovascular (CV) risk factor if the patient had eGFR < 60 mL/min/1.73 m^2^ [6]. Currently, it is recognized that CKD identified by either high uACR as well as low eGFR is a significant and independent risk factor for CVD [7]. Regular cardiovascular risk assessment should include evaluation of CKD, based on either low eGFR (<60 mL/min/1.73 m^2^) or increased albuminuria uACR (>30 mg/g). The recommendation form ESC 2021 Guidelines on CVD Prevention in Clinical Practice, which states that uACR and eGFR should also be evaluated in addition to blood pressure, cholesterol, and glucose to estimate an individual’s cardiovascular risk, have significant implications for both kidney health and cardiovascular risk screening. Any assessment of cardiovascular risk should include measurement of albuminuria (uACR) and serum creatinine (eGFR) [8].

The purpose of this study was to assess the prevalence of impaired kidney function in Polish patients with CCS based on their eGFR and albuminuria, especially in the subpopulation with eGFR > 60 mL/min/1.73 m^2^.

## 2. Patients and Methods

### 2.1. Study Population

The study was carried out in seven Polish centers, Bialystok, Bydgoszcz, Cracow, Katowice, Rzeszow, Warsaw, and Zamosc, in two periods, 2016–2018 and 2022–2023, in the framework of projects Polaspire [9] and Polaspire2, respectively [10].

### 2.2. Data Collection and Assays

During the study visit, questionnaires were used to gather the participants’ medical history information. A physical examination and laboratory assessment were performed on each research patient. After an overnight fasting, peripheral intravenous blood samples were taken. Random urine samples were used to assay uACR. All the samples were analyzed in the local laboratories of the participating hospitals. Anthropometric measurements, including weight, circumferences of the waist, abdomen, and hips, were taken. Following at least five minutes of sitting, the participants’ blood pressure (BP) was measured using the oscillometric method. A resting electrocardiography (ECG) was performed.

The group was classified according to Kidney Disease: Improving Global Outcomes (KDIGO): ≥90 (normal renal function); 60–89 (mild impairment of renal function (IRF)); 30–59 (moderate IRF); 15–29 (severe IRF); and <15 mL/min/1.73 m^2^ (end-stage IRF). eGFR was calculated using the 2009 Chronic Kidney Disease Epidemiology Collaboration (CKD-EPI) formula: for females with serum creatinine (SCr) ≤ 0.7 mg/dL: 144 × (SCr [mg/dL]/0.7)^(−0.329) × 0.993^Age [years] (×1.159 if black race); for females with SCr > 0.7 mg/dL: 144 × (SCr [mg/dL]/0.7)^(−1.209) × 0.993^Age [years] (× 1.159 if black race); for males with SCr ≤ 0.9 mg/dL: 141 × (SCr [mg/dL]/0.9)^(−0.411) × 0.993^Age [years] (×1.159 if black race); for males with SCr with >0.9: 141 × (SCr [mg/dL]/0.9)^(−1.209) × 0.993^Age [years] (×1.159 if black race) [11].

**Figure 1:**
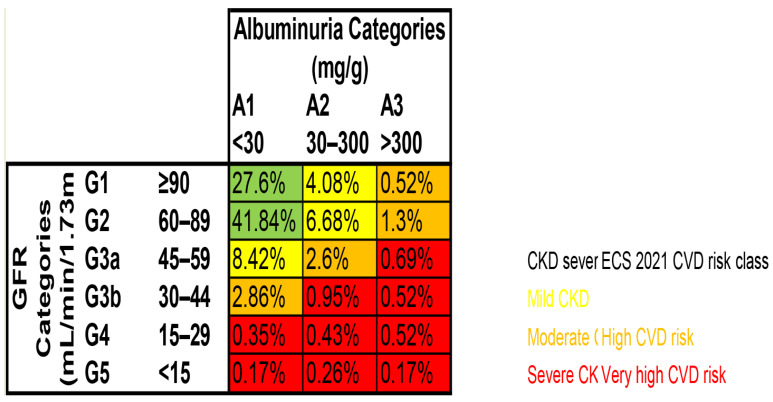
Classification of chronic renal disease (as defined by Renal Disease: Improving Global Outcomes in 2012) based on albuminuria and estimated GFR in all patients with CCS. N = 1152 N—population with known eGFR and albuminuria. Colors: green—preserved function; yellow—mild CKD; orange—moderate CKD (high CVD risk); red—severe CKD (very high CVD risk).

Based on the KDIGO classes, taking into account eGFR and uACR, we used three CVD risk classes (yellow, orange, and red). Green indicates preserved kidney function and was reclassified based on uACR measurement. The described change in CKD class after the assessment of albuminuria is based on the simultaneous assessment of eGFR and albuminuria concentration and reclassification of patients from a lower risk group to a higher risk group based on the increase in albuminuria concentration and, at the same time, preserved eGFR (Figure 1).

### 2.3. Ethical Issues

Ethical approval for this study was provided by the Ethics and Supervision Committee for Research on Humans and Animals at the Central Clinical Hospital of the Ministry of Internal Affairs and Administration in Warsaw (approval number: 76/2016, 16/2022), the Bioethics Committee of the Medical University of Silesia in Katowice (approval number: KNW/022/125/16, KNW/0022/KB1/125/16), the Bioethics Committee of the Jagiellonian University in Krakow (approval number: K/ZDS/006249, 1072.6120.94/2021), the Bioethics Committee of the Center for Postgraduate Medical Education (approval number: 72/2022, 129/2022), the Ethics Committee of the Medical University of Bialystok (approval number: R-I-002/323/2016, R-I-002/324/2016, APK.002.139.2020), and the Bioethics Committee of the Nicolaus Copernicus University in Torun (approval number: 164/2022).

The study was conducted in accordance with the Declaration of Helsinki.

All the participants provided written informed consent.

### 2.4. Statistical Analysis

Normality of distribution was verified using the Kolomogorow–Smirnow test. For non-normal distribution, continuous variables were shown as the median with the interquartile range (IQR) [first quartile–third quartile]. Continuous variables following other than normal were compared using the Mann–Whitney U test. Associations between the presence of eGFR < 60 mL/min/1.73 m^2^, CKD severity or prognosis change, and other clinical and biochemical variables were analyzed using simple and multiple logistic regression models adjusted for age, gender, and weight. The results of the multivariate analysis were presented as figures. ANOVA tests were used to compare variables between subgroups, and a post hoc Tukey’s Honest Significant Difference test was used to determine the difference between the groups. Frequency tables were used to summarize categorical variables. The results were regarded as statistically significant at the 0.05 level. All computations were performed using IBM SPSS Statistics 26.0 (Armonk, NY, USA).

## 3. Results

The study visit was performed on 1957 patients aged 25 to 80 years, 6 to 18 months after hospitalization due to coronary artery bypass grafting (CABG) 147 (7.51%) or elective percutaneous coronary intervention (PCI) 587 (29.99%) or acute myocardial infarction with ST-segment elevation (STEMI) 362 (18.5%) or acute myocardial infarction with non-ST-segment elevation (NSTEMI) 436 (22.28%) or unstable angina 395 (20.18%). Each center obtained its own Ethical Committee approval for the particular part of the study. With regard to 30 (1.5%) patients, there was no data concerning the cause of hospitalization during the indexed event. As many as 1152 patients had complete eGFR and uACR data. The study population is presented in Figure 1.

Table S1 (presented in Supplementary Material) contains the baseline characteristics of the study population. The median age was 67 (61–72) years, and 71.23% of participants were male. Overall, the median of eGFR was 80.21 (63.23–92.27), and the uACR was 7.26 (3.61–19.90).

Patients having an eGFR lower than 60 mL/min/1.73 m^2^ and greater than 60 mL/min/1.73 m^2^ are compared and presented in Table 1. The individuals with eGFR greater or equal than 60 mL/min/1.73 m^2^ were younger (*p* < 0.001) and heavier (*p* = 0.011). The BPd (*p* = 0.003) had a significant impact on the eGFR. There were no differences in waist circumference (*p* = 0.743), HR (*p* = 0.094), or BPs (*p* = 0.316). In the laboratory test, lower levels of NT-proBNP (*p* < 0.001), fasting glucose (*p* = 0.037), HbA1c (*p* = 0.001), and uACR (*p* < 0.001) were related with eGFR higher than 60 mL/min/1.73 m^2^. The eGFR was inversely associated with HDL concentration (*p* = 0.007) and positively with total cholesterol concentration (*p* = 0.020) but was not related with LDL.

Patients with albuminuria <30 were younger (*p* < 0.001) and also had lower HR (*p* < 0.001), lower BP (*p* = 0.003), and higher HDL concentration (*p* = 0.021) than patients with albuminuria 30–300 and >300. Furthermore, laboratory tests showed lower NT-proBNP (*p* < 0.001), fasting glucose (*p* < 0.001), and HbA1c (*p* < 0.001) concentrations in the category with albuminuria <30 compared to the category with albuminuria 30–300 and >300 (Table 2).

Patients with significant renal disease made up 30.52% of the sample; 19.18% had mild CKD. In 7.28% of cases, the CKD was moderate, and 4.06% of the study patients presented severe CKD. People with albuminuria over 300 have a high or very high risk of CVD. In 200 patients (Table 3), the determination of albuminuria increased the estimated CVD risk, indicating that the eGFR result is not enough to comprehensively determine a patient’s CVD risk. The albuminuria score demonstrates how these risks change depending on glomerular filtration rate damage and the presence of proteinuria. A detailed description of the albuminuria and eGFR categories is given in Figure 1.

The impact of the chronic renal disease on the cardiovascular risk with division of patients depending on previous unstable (myocardial infraction or unstable angina as index event) course of the disease is presented in Supplementary Material: Figures S1 and S2, and Table S2. The type of qualifying event is important in the assessment of CKD. Figures S1 and S2 show that a higher percentage of patients after planned CABG or PCI (32.84%) had not preserved renal function than patients after an acute coronary syndrome (28.84%). Moreover, the profile of these patients also differed significantly from each other, as detailed in Table S2.

Two patient groups, one whose CVD risk category was altered and the other whose risk did not change after examining albuminuria, are compared in Table 3. Older (*p* < 0.001) individuals with higher BPs (*p* = 0.008), BPd (*p* = 0.038), and HR (*p* < 0.001) were those who had a change in CVD risk, and hence the accurate assessment benefited from the additional measurement of albuminuria. The subjects with the change of CVD risk category had significantly elevated fasting glucose (*p* < 0.001) and HbA1c (*p* < 0.001), and lower HDL concentration (*p* = 0.001).

**Figure 2:**
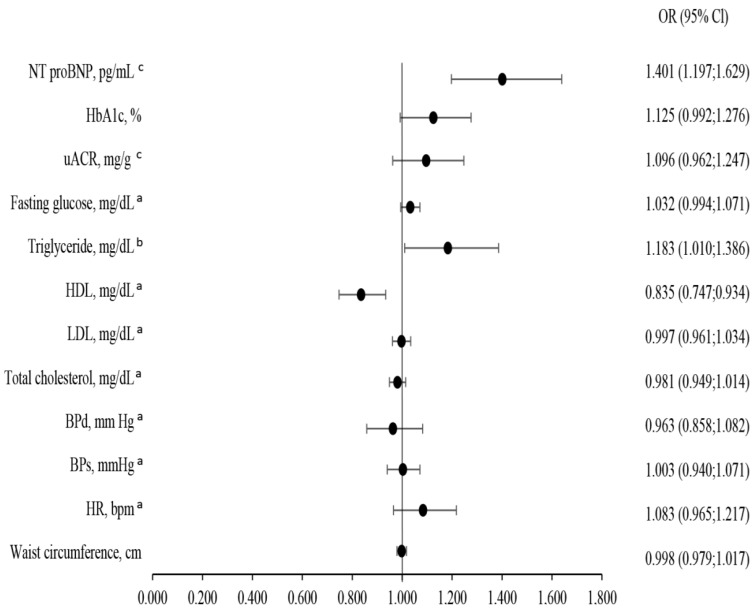
Association of clinical variables with eGFR < 60 mL/min/1.73 m^2^ adjusted for age, gender, and weight in multivariate logistic regression analysis. ^a^ Per 0.1 units; ^b^ Per 0.01 units; ^c^ Per 0.001 units. Abbreviations: see Table 1.

Figure 2 shows the adjusted odds of having eGFR < 60 mL/min/1.73 m^2^. In this subpopulation, the eGFR < 60 mL/min/1.73 m^2^ was associated inversely with HDL (*p* = 0.002) and positively with triglycerides (*p* = 0.037) and NT-proBNP concentrations (*p* < 0.001).

**Figure 3:**
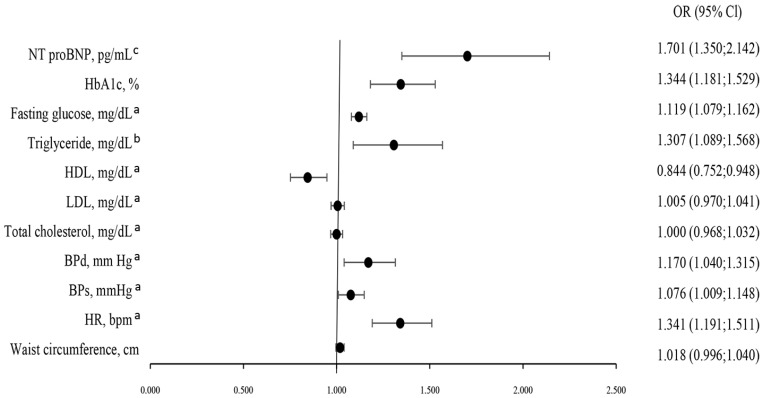
Multivariable (logistic regression analysis) predictors of mild, moderate, or severe CKD (adjusted for age, gender, and weight). ^a^ Per 0.1 units; ^b^ Per 0.01 units; ^c^ Per 0.001 units. Abbreviations: see Table 1.

The odds of the presence of severe CKD adjusted for age, gender, and weight is presented in Figure 3. BPs (*p* = 0.025), BPd (*p* = 0.009), HR (*p* < 0.001), and HDL (*p* = 0.004) were significantly associated with the incidence of CKD severity. The CKD severity also increased with raised triglyceride (*p* = 0.004), fasting glucose (*p* < 0.001), HbA1c (*p* < 0.001), and NT-proBNP (*p* < 0.001).

**Figure 4:**
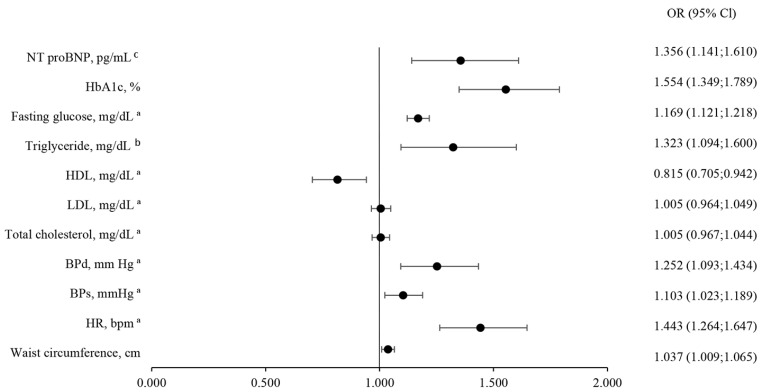
The odds ratio for reclassification of the CKD group after adding albuminuria to eGFR (adjusted for age, gender, and weight) using logistic regression analysis. ^a^ Per 0.1 units; ^b^ Per 0.01 units; ^c^ Per 0.001 units. Abbreviations: see Table 1.

Figure 4 presents the association of clinical variables with the odds of reclassification (increase) of CKD class after assessment of uACR. The multivariate analysis is adjusted for age, gender, and weight and presents variables which characterize patients in whom assessment of uACR is particularly important. The change in CKD class, which entails increased CVD risk, was correlated with BPs (*p* = 0.010), BPd (*p* = 0.001), HR (*p* < 0.001), and waist circumference (*p* = 0.009). Moreover, we observed a significant interaction between glucose metabolism, especially fasting glucose (*p* < 0.001), HbA1c (*p* < 0.001), and NT pro-BNP (*p* < 0.001) in the multivariate analysis. A significant positive association, particularly in HDL (*p* = 0.006) and triglyceride (*p* = 0.004), was observed in patients with changed CVD risk.

The numerical data of the above logistic regressions have been reposted in the Supplement (Figures S3 and S5).

## 4. Discussion

The prevalence of chronic kidney disease in the general global population is estimated at 10% [12]. In patients with chronic coronary syndrome, this number is higher. In our study, we demonstrated the prevalence of CKD (adjusted for albuminuria) in patients with CCS to be at 30.52% (mild: 19.18%; moderate: 7.28%; severe: 4.06%).

The available literature says that, considering only the criterion of eGFR, the prevalence of CKD in the CCS patient population ranges from 11.48% [13] to as high as 37.3%, depending on the utilized criteria [14]. Several studies showed that, after including albuminuria as a criterion, CKD may be diagnosed in about 30.9–39.2% [15,16] of patients with a history of CCS. In addition, Samaan [15] demonstrates in his paper the distribution of CKD prevalence by stage: mild: 25.5%; moderate: 3.6%; and severe: 1.7%. Considering that albuminuria increases the accuracy of assessment, our work focuses its evaluation on this important factor of uACR determination in patients with CCS. This analysis indicates that albuminuria has a significant impact on determining the prognosis, even when eGFR is already known. Even in a patient with preserved eGFR, the presence of albuminuria changes the risk group. Therefore, groups of patients are being identified at higher risk than initially thought based solely on eGFR measurement.

The parameters compiled in the study, such as blood pressure, glucose metabolism parameters, lipid profile, NT-proBNP, and waist circumference, are parameters associated with both CKD and CCS. We aimed to identify the patients in whom uACR assessment would have the biggest probability to alter the prognosis.

Previous studies based on the same study population presented the distribution of anti-protein drugs taken by patients (ACE inhibitors/ARB (65.1–90.3%)—depending on the qualifying event; SGLT inhibitors (12.6–14.5%)—depending on gender) [9,10].

### 4.1. Estimated GFR in Patients with CCS

The 2021 guideline recognizes CKD, diagnosed as a low eGFR (<60 mL/min/1.73 m^2^), as a CV risk factor [8]. Accordingly, the study compared groups whose cutoff was below 60 and greater than or equal to 60 (Table 1).

In our study, the lower eGFR was associated with a population whose majority were older men with lower weight. Considering the aforementioned criteria, we demonstrated that eGFR < 60 is positively correlated with higher triglyceride and lower HDL concentration, and negatively correlated with LDL concentration (Figure 2).

We have shown a positive association of high NT-proBNP with lower eGFR. This is somewhat related to the fact that this peptide is removed from the body by the kidneys. Therefore, with impaired kidney function, this marker accumulates in pathologically high amounts in the body.

### 4.2. Prevalence of Albuminuria

Albuminuria is associated with an approximately 40% increased risk of clinical CCS [17]. Despite this, the cardiology profession has underutilized and undervalued albuminuria as an indicator of disease risk [18].

In our study, albuminuria levels in the range of 30–300 (mg/g) affected 15% (N = 182) of patients; above 300 (mg/g), it was 3.72% (N = 45) (Figure 1). In total, this is almost 19% of the study population; kidney dysfunction would be underestimated if eGFR was performed alone. Our findings support the concept that revised CVD risk groups were estimated by performing uACR.

### 4.3. The Impact of Albuminuria Measurement on CVD Risk Groups

The concept of moderate and severe CKD has been expanded by including albuminuria in the definition. Thus, moderate CKD is no longer limited to those with eGFR 30–59 mL/min/1.73 m^2^, and severe CKD is no longer limited to GFR < 30 mL/min/1.73 m^2^. Moderate and severe CKD are now consistent with KDIGO’s global risk classes. KDIGO includes a combination of eGFR and uACR values, which are associated with high or very high CVD risk. CKD is now diagnosed not only based on a low GFR < 60 mL/min/1.73 m^2^ but also taking into consideration a high uACR (>30 mg/g) as a strong and independent risk factor for CVD [8]. We showed that the number of people with serious kidney disease was underestimated based on guidelines involving the uACR and eGFR and the table based on them. In our research, as many as 30.52% of patients had mild, moderate, or severe CKD (Table S1). After obtaining the albuminuria result, as many as 200 (17.35%) patients changed their CVD risk group to a higher one.

### 4.4. Predictors of CKD Severity

In this study, we showed that heart rate, systolic blood pressure, diastolic blood pressure, triglyceride and HDL concentration, glucose, HbA1c, and NT-proBNP were significantly associated with the severity of CKD in patients with CCS (Figure 3).

The available literature vaguely describes the association of risk factors with the severity of CKD in patients with CCS, so for the most part the data compiled are for the general population.

Hannan indicates that higher BPs was associated with a higher risk of kidney failure, which is consistent with ESC recommending a BPs target of <130/80 mm Hg in individuals with CKD [19,20].

This is also confirmed by Joo, who reports in favor of a lower BPs target for delaying CKD progression in patients with CKD [21]. The relationship between systolic blood pressure and CKD progression is also discussed in Kim’s paper, but, more than that, the authors point out that there is a significant association between CKD progression and glucose levels; as well as the significance for predicting outcomes being decreased in the progression of renal dysfunction, triglycerides, HDL, and LDL concentrations were well correlated with CVD [22].

Arnold, using multivariate analyses in his study, emphasizes that, regardless of diabetes status or stage of CKD, HbA1c levels exhibit a tight concentration-dependent connection that makes them a strong predictor of eGFR deterioration [23].

Albuminuria should especially be determined in elderly patients and those with hypertension, diabetes, autoimmune diseases, and cancer.

### 4.5. Study Limitations

The data included in our analysis are limited to a single urine sample collected for testing uACR. The examination required an effort on the part of the patient to attend; hence, a large number of dialysis patients and severe CKD patients are underrepresented. Moreover, some patients did not have results for uACR. Moreover, patients over 80 years were not included in the study. Also, the analyzed group is not representative for patients with a new diagnosis of CCS. We did not analyze complete individual data on the medications taken by individual patients. Furthermore, the study does not discuss the impact of diabetes and hypertension due to previous work published on the same group of patients. The absence of a complete cross-section of CCS patients in the study population introduces a potential for selection bias. Therefore, our conclusions should be confirmed in further studies.

## 5. Conclusions

(1) Based on our analysis, we can conclude that the determination of albuminuria increases the accuracy of chronic kidney disease risk assessment in CCS patients, which improves identification of patients at higher cardiovascular risk.

(2) The CCS population presents a high prevalence of CKD. It has been shown that there is a significant proportion of patients with albuminuria even with normal or nearly eGFR.

(3) BPs, BPd, HR, and biochemical parameters such as HDL concentration, triglyceride, fasting glucose, HbA1c, and NT-proBNP were significantly associated with the severity of CKD and therefore with the level of CVD risk. 

## Figures and Tables

**Table 1:** Study population characteristics based on eGFR.

	Study Population (*n* = 1957)			
Variables	All Study Population	Subjects with <60 (*n* = 359)	Subjects with ≥60 (*n* = 1414)	*p* Values
Age, years	67.00 (61.00–72.00)	72.00 (67.00–76.00)	65.00 (60.00–71.00)	<0.001
Gender, male	1394 (71.23)	216 (60.17)	1061 (75.04)	<0.001
Weight, kg	83.00 (73.00–94.00)	80.20 (71.00–92.00)	83.00 (74.00–94.00)	0.012
Waist circumference, cm	101.00 (94.00–110.00)	101.00 (93.00–110.00)	101.00 (94.00–109.00)	0.787
HR, bpm	68.00 (61.00–75.00)	69.00 (62.00–76.00)	67.00 (61.00–75.00)	0.159
BPs, mmHg	135.00 (122.00–150.00)	137.00 (120.25–150)	135.00 (123.00–150.00)	0.240
BPd, mmHg	80.00 (72.00–88.00)	80.00 (70.00–86.00)	80.00 (74.00–89.00)	0.022
eGFR, ml/min/1.73 m^2^	80.21 (63.23–92.27)	48.40 (39.84–55.04)	86.14 (74.73–94.76)	<0.001
Total cholesterol, mg/dL	158.55 (132.25–193.26)	153.52 (127.00–189.37)	158.55 (134.00–193.35)	0.037
LDL, mg/dL	89.09 (65.74–119.88)	86.76 (63.09–115.16)	89.17 (67.00–120.00)	0.179
HDL, mg/dL	44.66 (36.67–54.00)	42.90 (35.00–52.07)	45.00 (37.00–54.14)	0.020
Triglyceride, mg/dL	111.00 (84.00–157.00)	110.09 (86.00–168.50)	111.00 (84.00–155.88)	0.449
Fasting glucose, mg/dL	108.00 (95.40–124.20)	109.98 (95.92–129.60)	107.37 (95.40–123.06)	0.033
uACR, mg/g	7.26 (3.61–19.90)	13.36 (5.06–63.10)	6.74 (3.36–15.93)	0.235
HbA1c, %	5.90 (5.60–6.40)	6.00 (5.60–6.70)	5.90 (5.60–6.30)	0.004
NT proBNP, pg/mL	182.80 (93.28–432.18)	366.65 (164.85–1029.50)	162.80 (88.00–365.78)	<0.001
Diabetes, % (*n* = 1655)	675 (40.79)	173 (44.5)	450 (30.4)	<0.001
Hypertension, % (*n* = 1921)	1613 (83.97)	338 (86.9)	1147 (77.5)	<0.001

Data are presented as median (Q1–Q3) or *n* (%). Q1, quartile 1; Q3, quartile 3; BPd, diastolic blood pressure; bpm, beats per minute; BPs, systolic blood pressure; cm, centimeter; dL, deciliter; eGFR, estimated glomerular filtration rate; CKD, Chronic Kidney Disease; ECE, Epidemiology Collaboration Equation; g, gram; HbA1c, Glycated hemoglobin; HDL, High-Density Lipoprotein; HR, heart rate; kg, kilogram; LDL, Low-Density Lipoprotein; m^2^, square meter; mg, milligram; min, minute; mL, milliliter; mmHg, millimeters of mercury; NT-proBNP, N-terminal pro-brain natriuretic peptide; pg, picogram; uACR, urine albumin/creatinine ratio.

**Table 2:** Characteristics of the study population according to the level of albuminuria.

Variables		Albuminuria < 30	Albuminuria 30–300	Albuminuria > 300	*p* Value
		*n* = 994	*n* = 182	*n* = 45	
General information	Age, years	66.00 (60.00–72.00) ^a,b^	68.00 (62.75–74.00) ^a^	69.00 (67.00–75.00) ^b^	<0.001
	Gander, male	716 (72.03)	134 (73.63)	33 (73.33)	0.905
	Weight, kg	83.00 (74.08–94.00)	82.00 (71.80–93.60)	84.80 (75.85–97.75)	0.319
	Waist circumference, cm	102.00 (95.00–110.00) ^b^	101.00 (95.00–110.00)	106.00 (98.00–116.00) ^b^	0.034
	Heart rate, bpm	66.00 (60.00–73.50) ^a,b^	70.00 (63.00–79.25) ^a^	73.00 (65.00–80.50) ^b^	<0.001
	BPs, mmHg	133.00 (120.00–147.00) ^b^	137.00 (122.00–152.00)	142.00 (130.00–159.50) ^b^	0.003
	BPd, mmHg	80.00 (72.00–87.00)	80.00 (74.00–90.00)	81.00 (72.00–90.00)	0.067
Laboratory tests	Total cholesterol, mg/dL	160.31 (135.00–193.35)	151.29 (123.16–195.30)	166.00 (126.45–199.39)	0.828
	LDL, mg/dL	89.06 (66.78–121.01)	85.46 (63.00–111.67)	89.50 (59.84–128.30)	0.944
	HDL, mg/dL	44.00 (36.67–53.17)	40.86 (34.03–48.68)	37.04 (33.25–44.00)	0.021
	Triglyceride, mg/dL	110.00 (80.33–157.57)	110.71 (85.03–158.00)	141.12 (93.00–201.05)	0.039
	Fasting glucose, mg/dL	108.00 (95.40–122.01) ^a,b^	124.20 (106.20–149.40) ^a^	134.91 (105.80–164.34) ^b^	<0.001
	eGFR, mL/min/1.73 m^2^	82.86 (68.00–93.34) ^a,b^	74.73 (56.39–90.66) ^a,c^	59.64 (41.10–80.84) ^b,c^	<0.001
	uACR, mg/g	5.46 (3.00–10.30) ^b^	70.59 (45.43–131.09) ^c^	678.52 (441.31–1087.78) ^b,c^	<0.001
	HbA1c, %	5.90 (5.60–6.30) ^a,b^	6.10 (5.70–7.20) ^a^	6.40 (5.90–7.70) ^b^	<0.001
	NT proBNP, pg/mL	163.20 (83.96–367.30) ^a,b^	164.40 (147.78–1021.75) ^a,c^	729.00 (225.20–2260.50) ^b,c^	<0.001
Comorbidities	Diabetes, %	328 (31.1) ^a,b^	82 (46.3) ^a^	19 (57.6) ^b^	<0.001
	Hypertension, %	836 (79.4) ^a^	156 (88.1) ^a^	28 (84.8)	0.002

Data are presented as median (Q1–Q3) or *n* (%). Q1, quartile 1; Q3, quartile 3; comparisons variables between subgroups. The same letters in each row (^a^ between albuminuria < 30 and albuminuria 30–300; ^b^ between albuminuria < 30 and albuminuria > 300; ^c^ between albuminuria 30–300 and albuminuria > 300) represent significant differences at *p* < 0.05. Abbreviations: see Table 1.

**Table 3:** Study population characteristics based on the presence of chance of CVD risk class (excluding patients with eGFR < 30 due to the fact that they belong to the highest risk category and this will not change after ACR measurement).

Study Population (*n* = 1153)			
Variables	Patients in Whom Albuminuria Did Not Change CKD Classification (*n* = 930)	Patients with the Change of CKD Class After Assessment of Albuminuria (*n* = 200)	*p* Values
Age, years	66.00 (60.00–71.5)	68.00 (63.00–74.00)	<0.001
Gender, male	667 (71.72)	151 (75.50)	<0.001
Weight, kg	83.10 (74.00–94.00)	83.00 (72.75–94.15)	0.735
Waist circumference, cm	102.00 (95.00–110.00)	103.00 (95.25–111.38)	0.170
HR, bpm	66.00 (60.00–74.00)	71.00 (64.00–80.00)	<0.001
BPs, mmHg	133.00 (121.00–147.00)	139.00 (125.00–152.25)	0.008
BPd, mmHg	72.50 (80.00–87.00)	80.00 (74.00–90.00)	0.038
eGFR, mL/min/1.73 m^2^	83.13 (68.47–93.37)	74.90 (57.38–90.63)	<0.001
Total cholesterol, mg/dL	159.31 (135.26–193.35)	153.30 (121.5–193.88)	0.100
LDL, mg/dL	89.17 (66.56–120.83)	85.58 (60.83–112.14)	0.192
HDL, mg/dL	44.00 (36.67–53.00)	39.99 (34.03–48.31)	0.001
Triglyceride, mg/dL	110.00 (80.07–157.75)	112.48 (87.00–170.05)	0.168
Fasting glucose, mg/dL	108.00 (95.87–122.01)	124.54 (108.00–150.30)	<0.001
uACR, mg/g	5.43 (2.95–10.30)	87.64 (48.07–200.94)	<0.001
HbA1c, %	5.90 (5.60–6.30)	6.10 (5.70–7.48)	<0.001
NT proBNP, pg/mL	163.40 (84.46–367.10)	362.30 (151.70–1007.50)	<0.001
Diabetes, %	282 (30.3)	96 (48.0)	<0.023
Hypertension, %	741 (79.9)	175 (87.5)	<0.001

Abbreviations: see Table 1.

## Data Availability

The data presented in this study are available on request from the corresponding author due to privacy restrictions.

## Supplementary Materials

The following supporting information can be downloaded at: https://www.mdpi.com/article/10.3390/jcm14186607/s1, Figure S1: Classification of chronic renal disease (as defined by Renal Disease: Improving Global Outcomes in 2012) based on albuminuria and estimated GFR in the patients with CABG or elective PCI as index event. N = 734 N—population with known eGFR and albuminuria; Figure S2: Classification of chronic renal disease (as defined by Renal Disease: Improving Global Outcomes in 2012) based on albuminuria and estimated GFR in all patients in the CCS patients with history of acute coronary syndrome. N = 1193 N—population with known eGFR and albuminuria; Figure S3: The numerical data of the logistic regressions presented in Figure 2; Figure S4: The numerical data of the logistic regressions presented in Figure 3; Figure S5: The numerical data of the logistic regressions presented in Figure 4; Table S1: Baseline characteristics of the study population; Table S2: Comparison of a group of patients with or without a history of acute coronary syndrome.
