# Supplementary material for: Impairment of Kidney Function in Patients with Chronic Coronary Syndromes
Source: J Clin Med. 2025 Sep 19;14(18):6607. doi: 10.3390/jcm14186607 (PMC12470754; doi:10.3390/jcm14186607)
Supplement: Supplementary file 1 [file jcm-14-06607-s001.zip › Table S1.pdf]

Variables	Value (n=1957)
Age, years	67.00 (61.00-72.00)
Gender, male	1394 (71.23)
Weight, kg	83.00 (73.00-94.00)
Waist circumference, cm	101.00 (94.00-110.00)
HR, bpm	68.00 (61.00-75.00)
BPs, mmHg	135.00 (122.00-150.00)
BPd, mmHg	80.00 (72.00-88.00)
eGFR, ml/min/1.73m^2	80.21 (63.23-92.27)
Total cholesterol, mg/dL	158.55 (132.25-193.26)
LDL, mg/dL	89.09 (65.74-119.88)
HDL, mg/dL	44.66 (36.67-54.00)
Triglyceride, mg/dL	111.00 (84.00-157.00)
Fasting glucose, mg/dL	108.00 (95.40-124.20)
uACR, mg/g	7.26 (3.61-19.90)
HbA1c, %	5.90 (5.60-6.40)
NT proBNP, pg/mL	182.80 (93.28-432.18)
Diabetes, %	282 (30.3)
Hypertension, %	741 (79.9)
