# Supplementary material for: Impairment of Kidney Function in Patients with Chronic Coronary Syndromes
Source: J Clin Med. 2025 Sep 19;14(18):6607. doi: 10.3390/jcm14186607 (PMC12470754; doi:10.3390/jcm14186607)
Supplement: Supplementary file 1 [file jcm-14-06607-s001.zip › Table S2.pdf]

Study population (n=1957)			
Index event	CABG + PCI (n=734)	STEMI + NSTEMI + UA (n=1193)	p values
Age, years	68.00 (62-73)	66.00 (60-72)	<0.001
Gender, male	555 (75.61%)	839 (70.33%)	<0.001
Weight, kg	84.00 (74.70-95.00)	82.00 (72.00-93.00)	0.018
Waist circumference, cm	102.00 (96.00-111.00)	100.00 (93.00-109.00)	<0.001
HR, bpm	67.00 (60.00-75.00)	68.00 (62.00-75.00)	0.149
BPs, mmHg	135.50 (122.00-150.00)	135.00 (122.00-149.00)	0.447
BPd, mmHg	80.00 (72.50-87.50)	80.00 (72.00-89.00)	0.222
eGFR, ml/min/1.73m²	79.55 (62.16-91.29)	80.88 (63.95-93.08)	0.036
Total cholesterol, mg/dL	153.00 (130.24-177.00)	164.32 (134.42-201.08)	<0.001
LDL, mg/dL	84.80 (61.87-104.40)	93.19 (69.00-130.41)	<0.001
HDL, mg/dL	45.61 (37.05-55.01)	43.85 (36.10-52.64)	0.006
Triglyceride, mg/dL	107.00 (80.60-148.00)	112.48 (86.00-162.08)	0.057
Fasting glucose, mg/dL	111.06 (97.02-127.80)	105.89 (95.04-122.40)	<0.001
uACR, mg/g	7.92 (4.00-22.15)	6.76 (3.32-17.01)	0.010
HbA1c, %	6.00 (5.70-6.50)	5.80 (5.50-6.30)	<0.001
NT proBNP, pg/mL	185.05 (96.70-518.00)	179.50 (91.60-412.18)	0.216
