# Supplementary figures and images for: Impairment of Kidney Function in Patients with Chronic Coronary Syndromes
Source: J Clin Med. 2025 Sep 19;14(18):6607. doi: 10.3390/jcm14186607 (PMC12470754; doi:10.3390/jcm14186607)

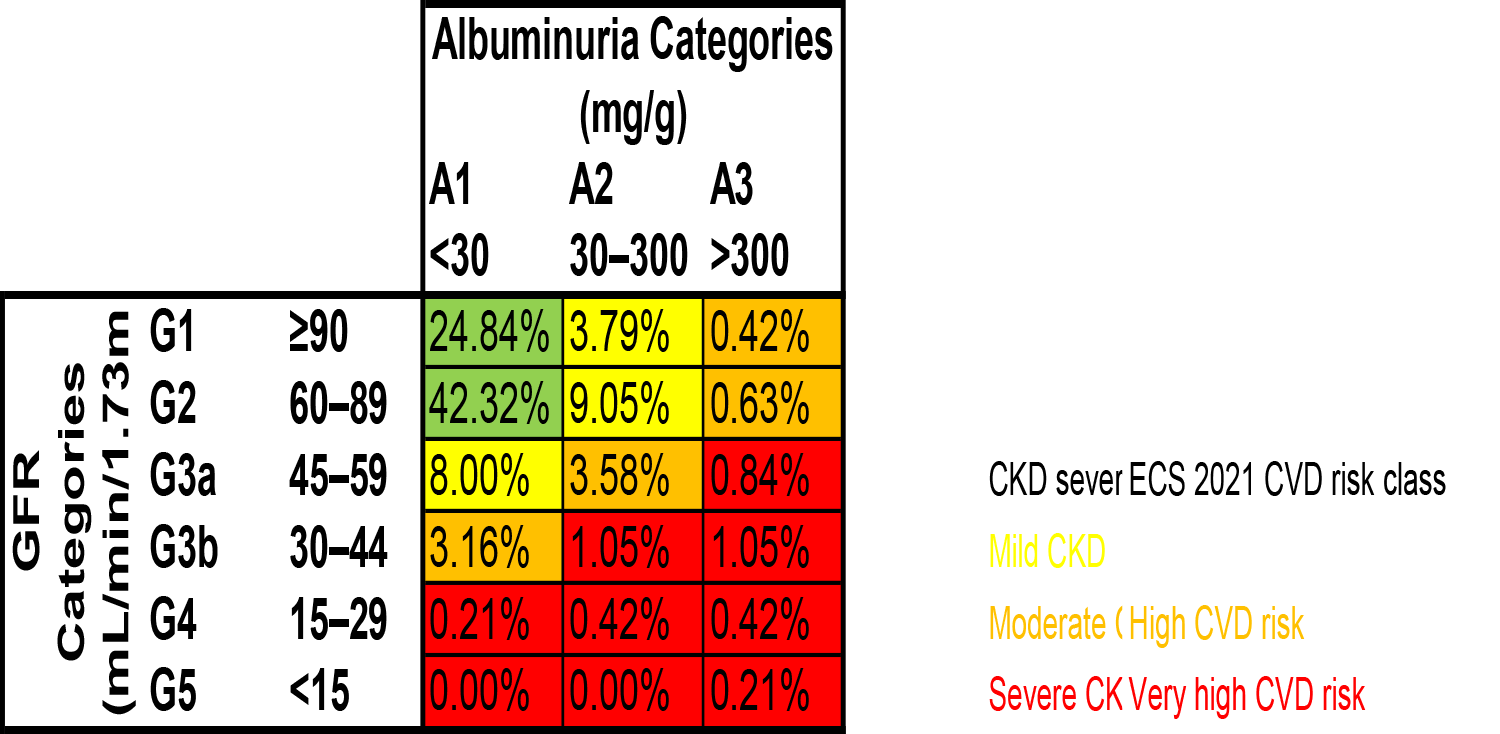

Supplement: Supplementary file 1 [file jcm-14-06607-s001.zip › Figure S1.tif]

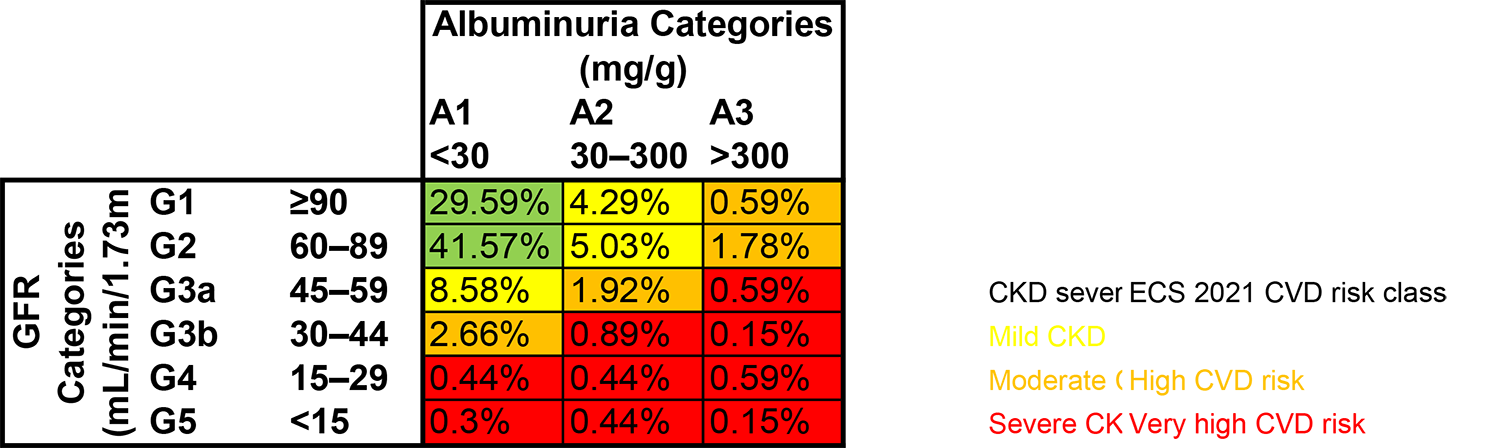

Supplement: Supplementary file 1 [file jcm-14-06607-s001.zip › Figure S2.tif]

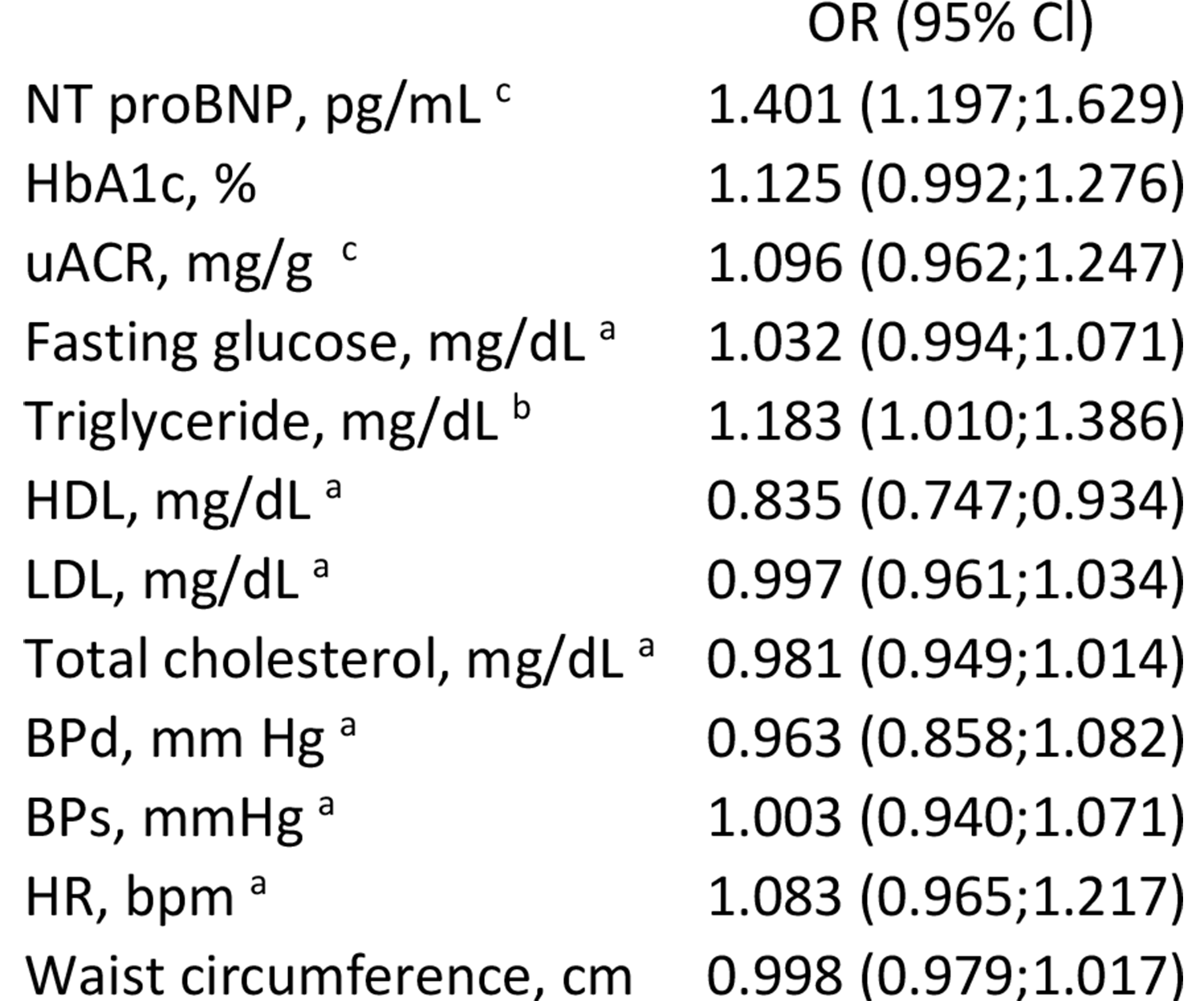

Supplement: Supplementary file 1 [file jcm-14-06607-s001.zip › Figure S3.tif]

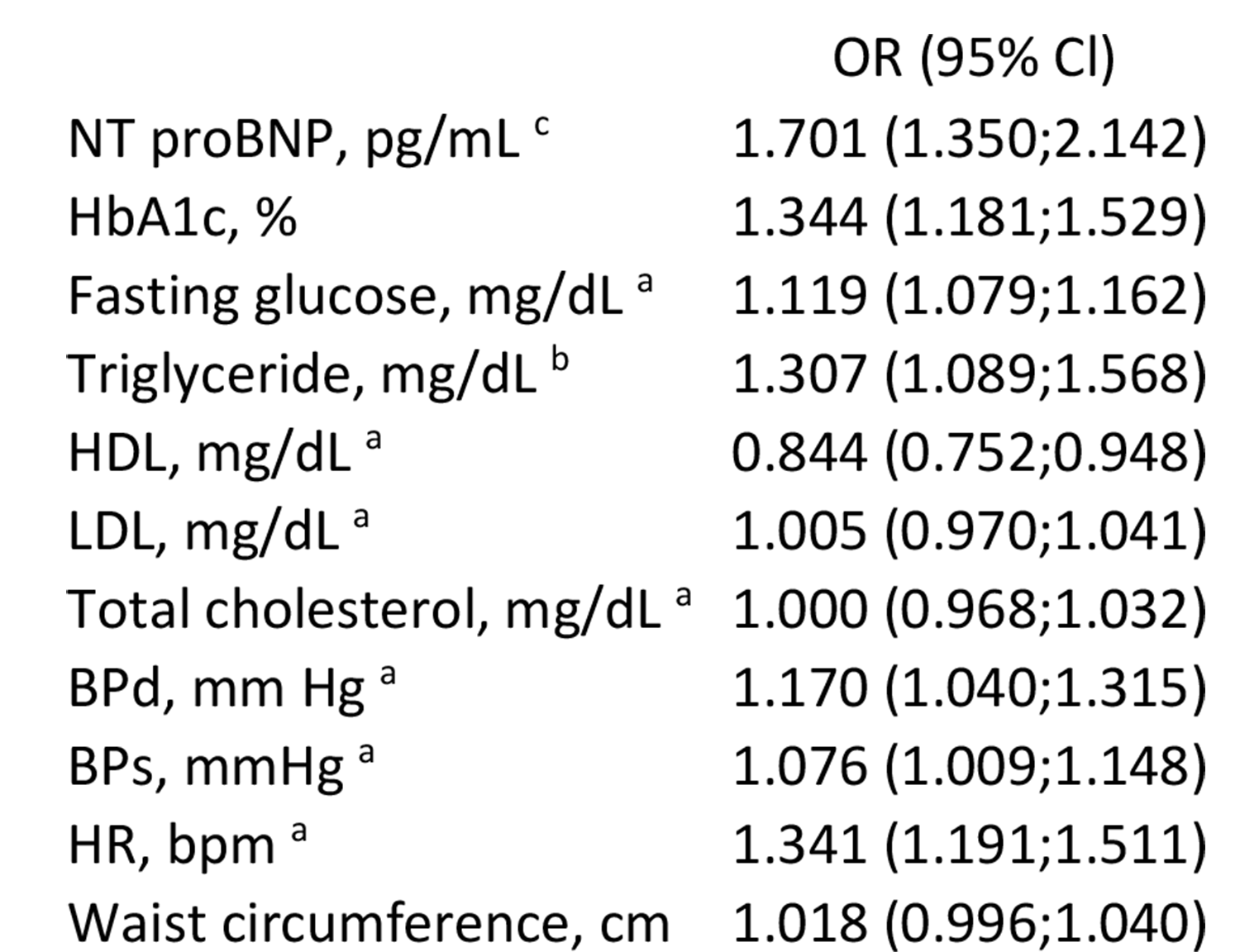

Supplement: Supplementary file 1 [file jcm-14-06607-s001.zip › Figure S4.tif]

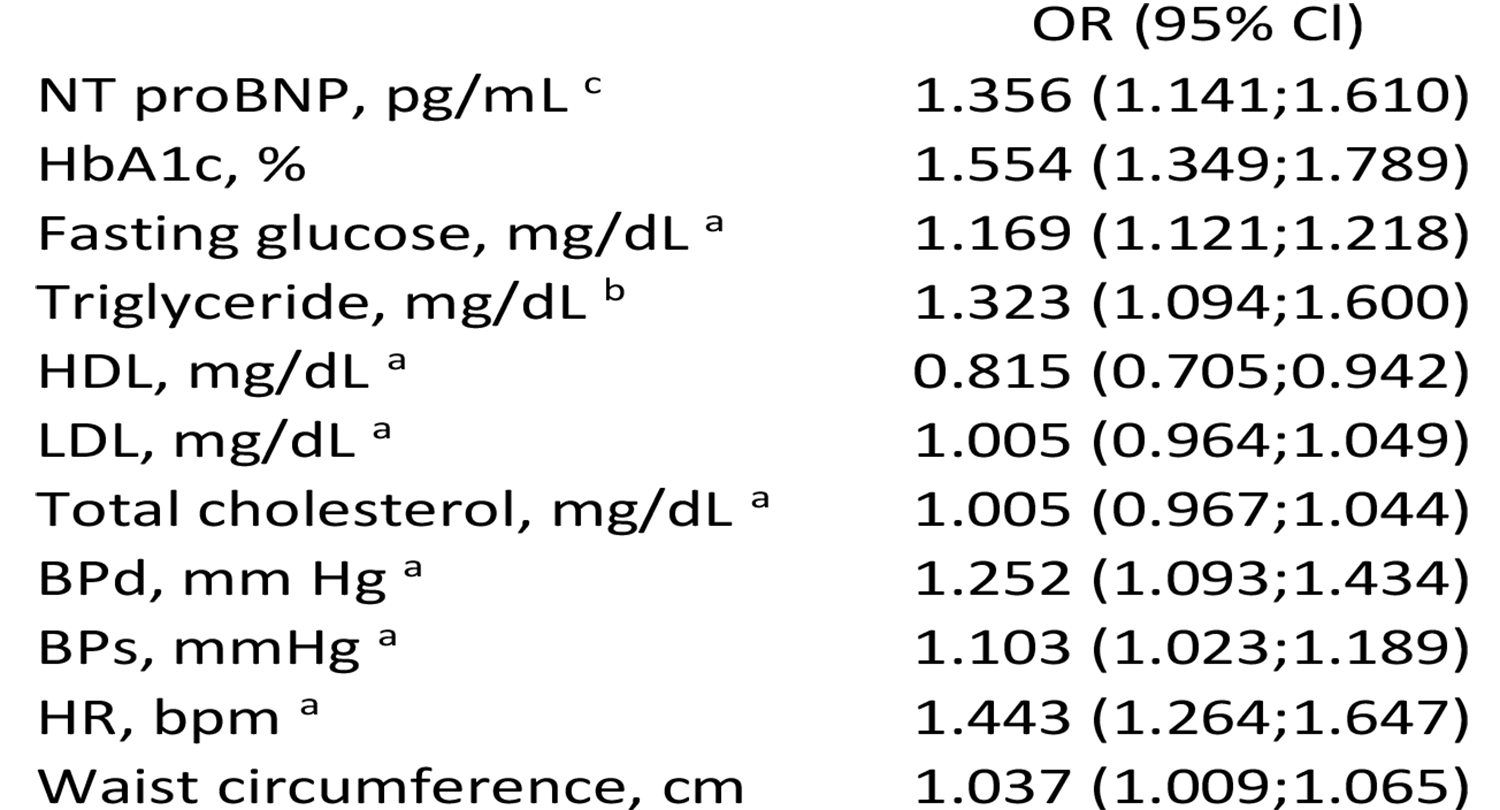

Supplement: Supplementary file 1 [file jcm-14-06607-s001.zip › Figure S5.tif]
